# Understanding the molecular mechanisms driving metastasis

**DOI:** 10.1002/1878-0261.12024

**Published:** 2017-01-09

**Authors:** Joan Massagué, Eduard Batlle, Roger R. Gomis

**Affiliations:** ^1^ Cancer Biology and Genetics Program Memorial Sloan Kettering Cancer Center New York NY USA; ^2^ Oncology Program Institute for Research in Biomedicine (IRB Barcelona) The Barcelona Institute of Science and Technology Spain; ^3^ ICREA Institució Catalana de Recerca i Estudis Avançats Barcelona Spain

Metastasis continues to be a lethal hallmark of cancer, with most patients dying as a result of the dissemination of the disease to foreign organs rather than as a consequence of the primary tumor. Malignant cells spread from the primary tumor to distant sites, where they resist conventional treatments, proliferate, and cause failure of vital organs. Systemic dissection of the molecular, cellular, genetic, and clinical mechanisms underlying metastatic progression is necessary for the development of new diagnostic and therapeutic strategies to prevent and treat metastases.

The aim of the systemic therapy that is given after tumor removal was to prevent metastatic relapse. However, the current pharmacological arsenal used in the adjuvant setting (chemotherapy) targets growing/proliferating tumor cells rather than metastasis. Preventing metastasis in high‐risk patients would be far better than having to treat them. Unfortunately, when tested, the few approved metastasis stroma‐modifying drugs (bisphosphonates, zometa, or anti‐RANKL antibody, denosumab) have yielded inconclusive results to date in the preventive adjuvant setting (Coleman *et al*., 2011, 2014; Smith *et al*., 2015) despite their clinical use to control bone metastasis morbidity (skeletal‐related events, pain, etc.). Therefore, the standard of care does not currently mandate any agent to prevent bone metastasis.

Different cancer types show distinct metastatic organ tropism. In addition, although steps in the metastatic cascade are part of a continuous biological sequence, their acquisition may vary from one tumor type to another (Nguyen *et al*., 2009). The classical simplification of metastasis into an orderly sequence of basic steps—local invasion, intravasation, survival in circulation, extravasation, and colonization—has helped to rationalize the complex set of biological properties required for a particular malignancy to progress toward overt metastatic disease (Gupta and Massagué, 2006). However, the kinetics of the metastasis and, in particular, the mechanisms that regulate tissue‐specific metastasis remain poorly understood and the latter are the focus of this proposal. The slow progression of certain subtypes of cancer under the distinct selective conditions present in various tissues gives rise to metastatic speciation. To metastasize, cancer cells must orchestrate diverse cellular functions to overcome the difficulties of the metastatic cascade. These functions are not only limited to cell‐autonomous traits but are also highly dependent on the interaction of the metastatic cell with the tumor and host stroma (Obenauf and Massagué, 2015). In some cases, several functions are required to implement a single step, whereas others may influence multiple ones. This speciation is reflected by the distinct kinetics of cancer relapse to different sites in the same patient and by the coexistence of malignant cells that differ in organ tropism in patient‐derived samples (Baccelli *et al*., 2013; Bos *et al*., 2009; Lu *et al*., 2009, 2011; Pavlovic *et al*., 2015; Urosevic *et al*., 2014).

In this current series of reviews, we aimed to provide a global view on the different aspects that may govern the metastatic cascade. We focused on features that have recently captured the attention of the field and may drive the avenues of important findings in the near future. To this end, special attention has been directed toward mechanisms of cancer cell migration and invasion as they are central for metastatic dissemination and may depend on regulators controlling cellular plasticity. Building on this concept, we aim to cover how epithelial‐to‐mesenchymal transition (EMT) program contributes to such plasticity. A process that although transitory has recently been proposed to go beyond the simple acquisition of motility features but also relates to nonproliferative or quiescent state of circulating and disseminated tumor cells. Next, we aimed to cover the advances in circulating and disseminated tumor cell biology as well as on dormancy. Metastatic dormancy may explain why metastasis occurs years or even decades after primary tumor resection. Interestingly, clones expressing stable traits can be identify and are responsible of such extended latency periods but are difficult to be reconciled within the endowed mutational and genomic plasticity present in cancer cells. To this end, focus was placed on epigenetic determinants that may support metastasis. Finally, attention was directed to the growing evidence on the central role of the stroma in defining cancer metastasis.
